# Supplementary material for: Content-rich biological network constructed by mining PubMed abstracts
Source: BMC Bioinformatics. 2004 Oct 8;5:147. doi: 10.1186/1471-2105-5-147 (PMC528731; doi:10.1186/1471-2105-5-147)
Supplement: Additional File 2 — The original results of the above study (non-essential files are deleted to keep the file size under the limit set by BMC bioinformatics). [file 1471-2105-5-147-S2.bz2 › chilibotAdditionalFile2/dip10/434ID7622448E10379/html/CCL4_CCR3.html]

 


 **CCL4** and **CCR3** 
  
Found 13 abstracts in PubMed, retrieved 10.  
 

 What does Google say? 
 PDF only 
| .edu only 

---

**Interactive relationship** (e.g. stimulation, inhibition, etc)

**Non-interactive relationship** (e.g. studied together, co-existance, homology, etc.)

- The most active chemokines for resting Th IL 10, in terms of calcium mobilization and in vitro migration, were in order of potency CCL2 monocyte chemoattractant protein 1, CCR2 ligand,  **CCL4**  macrophage inflammatory protein 1beta, CCR5 ligand, CCL3 macrophage inflammatory protein 1alpha, CCR1 5 ligand, CCL17 thymus and activation regulated chemokine, CCR4 ligand, CCL1 I 309, CCR8 ligand, CXCL12 stromal derived factor 1, CXCR4, and CCL11 eotaxin,  **CCR3**  ligand.  Ref: 11145678 J Immunol, 2001
- In this study, the expression of  **CCR3** , CCR5, CXCR3, and CXCR4 were analyzed by flow cytometry in three distinct in vitro models of Th1 Th2 polarization, activated naive and memory T cells, and T cell clones, in which the intracellular synthesis of interferon gamma IFN gamma and interleukin 4 IL 4 and the surface expression of CD30 and LAG  [ **CCL4** ]  3 were also assessed.  Ref: 10331500 J Leukoc Biol, 1999
- CC CKR3  [ **CCR3** ]  also bound MCP 1 with lower affinity, but did not bind MIP 1 alpha or MIP 1 beta  [ **CCL4** ] .  Ref: 8642344 J Exp Med, 1996
